# The Bladder Is a Novel Target of Developmental Polychlorinated Biphenyl Exposure Linked to Increased Inflammatory Cells in the Bladder of Young Mice

**DOI:** 10.3390/toxics9090214

**Published:** 2021-09-08

**Authors:** Conner L. Kennedy, Audrey Spiegelhoff, Kathy Wang, Thomas Lavery, Alexandra Nunez, Robbie Manuel, Lauren Hillers-Ziemer, Lisa M. Arendt, Kimberly P. Keil Stietz

**Affiliations:** Department of Comparative Biosciences, School of Veterinary Medicine, University of Wisconsin-Madison, Madison, WI 53706, USA; clkennedy3@wisc.edu (C.L.K.); aspiegelhoff@wisc.edu (A.S.); kwang399@wisc.edu (K.W.); tlavery@wisc.edu (T.L.); asnunez@wisc.edu (A.N.); rmanuel@wisc.edu (R.M.); lauren.ziemer@nih.gov (L.H.-Z.); lmarendt@wisc.edu (L.M.A.)

**Keywords:** lower urinary tract, bladder, inflammation, POPs, developmental basis of adult disease

## Abstract

Bladder inflammation is associated with several lower urinary tract symptoms that greatly reduce quality of life, yet contributing factors are not completely understood. Environmental chemicals are plausible mediators of inflammatory reactions within the bladder. Here, we examine whether developmental exposure to polychlorinated biphenyls (PCBs) leads to changes in immune cells within the bladder of young mice. Female mice were exposed to an environmentally relevant mixture of PCBs through gestation and lactation, and bladders were collected from offspring at postnatal day (P) 28–31. We identify several dose- and sex-dependent PCB effects in the bladder. The lowest concentration of PCB (0.1 mg/kg/d) increased CD45+ hematolymphoid immune cells in both sexes. While PCBs had no effect on CD79b+ B cells or CD3+ T cells, PCBs (0.1 mg/kg/d) did increase F4/80+ macrophages particularly in female bladder. Collagen density was also examined to determine whether inflammatory events coincide with changes in the stromal extracellular matrix. PCBs (0.1 mg/kg/d) decreased collagen density in female bladder compared to control. PCBs also increased the number of cells undergoing cell division predominantly in male bladder. These results implicate perturbations to the immune system in relation to PCB effects on the bladder. Future study to define the underlying mechanisms could help understand how environmental factors can be risk factors for lower urinary tract symptoms.

## 1. Introduction

Lower urinary tract symptoms (LUTS) greatly impact quality of life. Patients seeking medical attention for these symptoms represent a significant health care cost [1,2]. LUTS encompass a diverse range of both storage and voiding dysfunction; symptoms can range from obstruction, weak stream or difficulty urinating to increased frequency, urgency, incontinence, and overactive bladder. LUTS are often treated symptomatically because the underlying etiology is not completely understood and likely multifactorial. A better understanding of causative agents may lead to more beneficial therapies and improvement in quality of life for patients.

Bladder inflammation, also termed cystitis, is one major cause of bladder dysfunction [3]. There are known factors which can give rise to bladder inflammation including infection (typically acute inflammation), genetics, autoimmunity and dietary influences [3,4]. Consequences of these factors can lead to states of chronic inflammation, which can induce fibrosis and continuation or worsening of symptoms such as pain, urgency and frequency [3,5,6]. However, a clear factor contributing to bladder inflammation is not always evident and could reside in the environment. Chemicals are capable of eliciting bladder inflammation, for example, cyclophosphamide is commonly used in rodent models to study acute and chronic interstitial cystitis phenotypes [7]. Despite the use of chemicals to model bladder inflammation in rodents, whether exposure to ubiquitous environmental chemicals such as polychlorinated biphenyls (PCBs) can contribute to a state of chronic bladder inflammation, fibrosis and bladder dysfunction is understudied.

PCBs are a class of persistent organic pollutants that continue to pose a risk to human health. PCBs are implicated as risk factors for developing neurodevelopmental disorders (NDDs), which often have comorbid symptoms of bladder dysfunction [8]. PCBs have also been linked to deleterious changes in other health outcomes such as reproductive and immune function [9,10,11,12,13,14,15,16,17,18]. Detectable levels of PCBs are present in serum and tissue samples from humans [9,19] and livestock [20], as well as commercial milk [20,21], air [22,23] and water [24]. While the manufacture of PCBs is banned, exposure continues due to persistence of PCBs in the environment (legacy sources), as well as release of contemporary PCBs as unintentional byproducts during manufacturing processes such as paint pigment production [25,26]. Contemporary PCBs have been found to make up a large portion of detected PCBs in recent samples from humans and livestock [20,27,28]. Contemporary PCBs tend to be lower-chlorinated congeners and were not necessarily produced as part of manufactured (Aroclor) PCB mixtures prior to the ban [29,30]. Since some of these contemporary PCBs were not part of legacy PCB mixtures, less is known about their effects on long term health outcomes. Here, we use the Markers of Autism Risk in Babies Learning Early Signs (MARBLES) PCB mixture which mimics the proportion of the top PCB congeners detected in the serum of women at risk of having a child with a neurodevelopmental disorder [28,31]. This mixture not only replicates an environmentally relevant mixture found in the human population, but also consists of legacy higher-chlorinated PCBs in addition to contemporary lower chlorinated PCBs such as PCB 11 [28,32].

PCBs are known to have deleterious effects on the proper function of the immune system. Several studies indicate deficits in the adaptive immune response and immunotoxicity in adults and adolescents [15,16,33,34,35,36,37]. There is also evidence that innate immunity is influenced by PCB exposure, with increases in inflammatory cytokines often observed as indices of immune activation [17,38,39]. In a follow up of Yusho patients who experienced high exposure to PCBs via contaminated food in 1968, plasma concentrations of several cytokines were higher in exposed patients compared to controls more than 30 years after the initial incident [40]. In line with epidemiological data, in animal models, PCBs have been shown to alter proinflammatory and profibrotic markers in serum, liver, and brain [41,42,43]. Recent animal studies have modeled environmentally relevant concentrations of PCBs using the MARBLES PCB mixture. In mice, developmental exposure to MARBLES PCB results in offspring which display alterations in inflammatory markers in the intestine which coincide with changes in intestinal physiology and the microbiota [44]. In addition, developmental exposure to MARBLES PCB leads to elevated levels of serum cytokines and chemokines in juvenile offspring [45]. While PCB exposures are linked to changes in circulating cytokines, less is known regarding the inflammatory signature within tissues such as the bladder.

To expand upon the evidence that PCBs can increase serum cytokine in juvenile mice following developmental exposure, we sought to determine whether PCBs alter inflammation in other organs of interest such as the bladder. We focus on the bladder as we have previously shown that PCBs are not only detected in mouse bladder following developmental exposure, but that PCBs lead to changes in neuromorphology which are correlated with increased mast cell numbers [32]. Here, we test the hypothesis that developmental exposure to the environmentally relevant MARBLES PCB mixture results in increased immune cells within the bladder of juvenile male and female mice and test whether these cells are associated with changes to the extracellular matrix collagen density.

## 2. Materials and Methods

### 2.1. Animals

All procedures involving animals were conducted in accordance with the NIH Guide for the Care and Use of Laboratory Animals and were approved by the University of California-Davis Animal Care and Use Committee (#18853 and 20584; date of approval: 8-5-15 and 8-3-18 respectively). Wild-type mice of 75% C57BL/6J/25% SVJ129 genetic background (Jackson Labs, Sacramento, CA, USA), were used in this study and were collected as part of a larger study as described previously [32,45]. All mice were housed in clear plastic cages containing corn cob bedding and maintained on a 12 h light and dark cycle at 22 ± 2 °C. Feed (Diet 5058, LabDiet, St. Louis, MO, USA) and water were available ad libitum.

### 2.2. Developmental PCB Exposures

We have previously described the MARBLES PCB mixture and doses selected, 0.1, 1 and 6 mg/kg body weight/day, which result in PCB levels in offspring tissues within ranges observed in humans and do not interfere with reproductive outcomes of the dam [32,45,46]. The proportion of individual PCB congeners in the MARBLES PCB mixture mimics the proportion of the top 12 PCB congeners identified in pregnant women at risk of having a child with a neurodevelopmental disorder [18,28,31]. PCBs were synthesized and authenticated by the Synthesis Core of the University of Iowa Superfund Research Program with >99% purity as reported previously [28]. PCBs were dissolved in organic peanut oil (Spectrum Organic Products, LLC, Melville, NY, USA), and mixed into organic peanut butter (Trader Joe’s, Monrovia, CA, USA). PCB doses were measured on a weigh boat and delivered to mouse cages for oral consumption. Organic peanut oil dissolved in peanut butter and without PCBs served as the dosing control (0 mg/kg). Female mice were dosed daily beginning two weeks prior to start of mating and continuing through pregnancy and lactation (through postnatal day 21). Adult male breeders were paired with female mice until a visible copulation plug was seen or until mice steadily gained weight indicative of pregnancy. Male and female offspring were weaned at P21 and group housed with same sex and dose littermates. Mice were euthanized via CO2 prior to collection of tissues at P28–31.

### 2.3. Immunohistochemistry

Bladders were processed for immunohistochemistry as described previously [32]. Immunofluorescence was performed on bladder sections essentially as described [47]. Briefly, slides were deparaffinized in xylene and rehydrated through a series of graded ethanols. Antigen retrieval was performed in citrate buffer (0.01 M, pH 6.00, 20 min at 50% microwave power). Sections were blocked for 1 h in blocking buffer containing 1% blocking solution, 5% goat serum (16210064, Fisher, Waltham, MA, USA) and 1% BSA fraction V (80055-682, VWR, Radnor, PA) in tris-buffered saline (TBS, 0.2 M Tris-HCl (Fisher BP153-1), 1.5 M NaCl (Fisher BP358-212). Blocking solution was prepared as a stock solution consisting of 10% Blocking reagent (501003304, Fisher) dissolved in 100 mM maleic acid (S25415, Fisher) and 150 mM NaCl (721016, Fisher) pH 7.5. Sections were incubated with primary antibodies listed in Table 1 overnight at 4 °C. Following TBS washes, secondary antibodies listed in Table 1 were applied to tissues for 1 h at room temperature. Secondary antibody was removed and 4′,6-diamidino-2-phenylindole, dilactate solution 300 nM (DAPI) (IC15757401, VWR) was applied for 5 min to counterstain nuclei. Slides were mounted in anti-fade mounting medium (90% glycerol (G33500, Fisher), 0.2% n-propyl gallate (AAA1087722, Fisher), and phosphate-buffered saline (SH3001304, Fisher)) and cover-slipped. For non-fluorescent immunohistochemistry the same protocol as above was followed with the addition of incubating slides in 0.5% hydrogen peroxide (H325500, Fisher) for 20 min prior to antigen retrieval, and for F4/80, sections were blocked in 5% fish gelatin (G7765, Sigma-Aldrich, St. Louis, MO, USA) for 1 h at room temperature. For CD3 and F4/80, after removal of secondary antibody, sections were incubated in ABC reagent (Vectastain Elite HRP ABC kit, PK-6100, Vector Laboratories) followed by DAB peroxidase substrate kit (SK-4100) for development according to manufacturer’s instructions. For CD79b, following removal of primary antibody, the ImmPRESS^®^ HRO Horse Anti-Rabbit IgG Polymer Detection Kit, Peroxidase (MP-7401, Vector Laboratories) was used per manufacturer’s instructions. Sections were counterstained for 30 sec with hematoxylin (6765001, Fisher), dehydrated in graded ethanol followed by xylene and mounted with Permount (SP15100, Fisher). Slides were imaged using an Eclipse E600 or Eclipse Ci compound microscope (Nikon Instruments Inc., Melville, NY, USA) with a Photometrics Dyno CCD camera or DS Ri2 camera (Nikon Instruments Inc.) interfaced to NIS elements imaging software (Nikon Instruments Inc.) or a semi-automated BZ-X710 digital microscope and stitching software (Keyence, Itasca, IL, USA). Cell counts were performed in Image J (Version 1.52a) using the cell counter tool in a blinded manner. Cell counts were either normalized to total cells, total stromal cells, or total epithelial cells, or normalized to total bladder area as indicated in each figure. An *n* = 4–6 bladders per treatment group were used in all analyses.

### 2.4. Collagen Quantification

Picrosirius red staining (PSR) staining was used to determine collagen density as described previously [48]. Images were captured from the red (PSR) and green (autofluorescent) channels and ImageJ was used to process images. Briefly, the green channel (autofluorescence) was subtracted from the red channel (collagen) using the Image Calculator feature of ImageJ. The image was then made binary, using the freehand selection tool a region of interest was drawn around the bladder stroma by an individual blinded to treatment conditions. The total area of this region of interest was determined using the Analyze-Measure feature. Once total area was established, the analyze particles feature was used to determine collagen pixel density within the region. Collagen area/total area was used to determine collagen density.

### 2.5. Statistics

Normality of data was assessed using the Kolmogorov–Smirnov or D’Agostino–Pearson omnibus (K2) test within GraphPad Prism 8. If data failed to pass normality, transformation (log or square root) was applied to restore normality. Two-way ANOVA followed by Tukey’s multiple comparisons tests were used to determine differences between or among treatment groups with *p* values ≤ 0.05 considered significant. N values for each endpoint are indicated in figure legends. Significant main effects of two-way ANOVAs are indicated in each figure in addition to post hoc comparisons indicated by asterisks and bars.

## 3. Results

### 3.1. Developmental PCB Exposure Increases Inflammatory Cells in the Bladder

**Figure 1:**
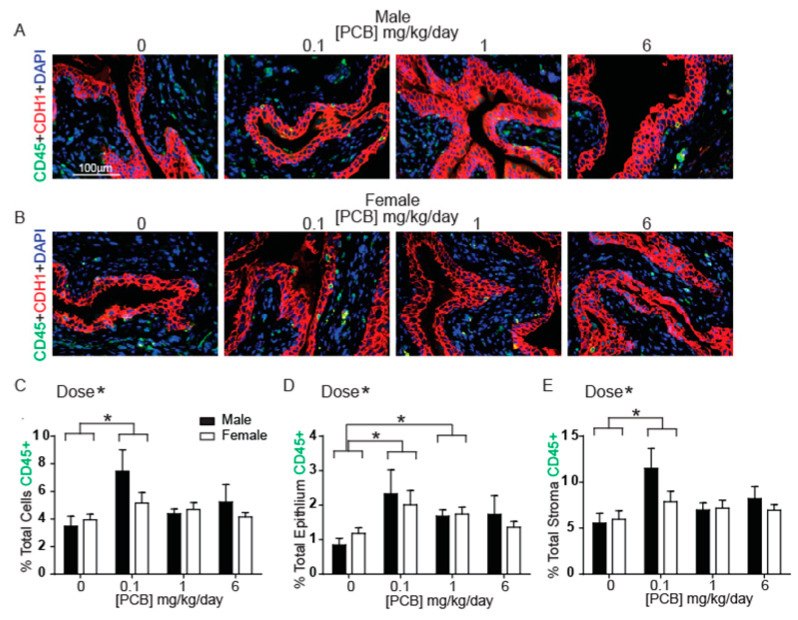
PCBs increase CD45-positive immune cells in bladder of developmentally exposed mice. Mice were exposed to PCBs via maternal diet through gestation and lactation and bladders collected from young male and female offspring at postnatal day (P) 28–31 for immunohistochemistry. Representative images of (**A**) male and (**B**) female mouse bladders at each PCB treatment group incubated with antibodies targeting CD45 (green) to label immune cells, e-cadherin (CDH1, red) to label all epithelium and DAPI (blue) to stain nuclei. Quantification of (**C**) the percent of total cells CD45 positive, (**D**) the percent of total epithelial cells CD45 positive and (**E**) the percent of total stromal cells CD45 positive. Results are the mean ± SEM, *n* = 4–6 bladders per treatment group, up to 3 images per bladder were averaged for final value. Significant differences at *p* < 0.05 are indicated by asterisk or asterisk and bars, as determined using two-way ANOVA followed by Tukey’s multiple comparisons tests.

Since PCBs cause inflammation in many tissues [38,44,49,50], and bladder inflammation can be a driver of LUTS [3], we tested and confirmed that mice developmentally (in utero and via lactation) exposed to MARBLES PCB develop low-grade bladder inflammation as young adults. We first chose to examine hematolymphoid cells defined as those positive for protein tyrosine phosphate receptor type C (PTPRC, CD45). There was a significant overall main effect of PCB dose on the percentage of CD45-positive hematolymphoid immune cells within the bladder, driven by an increase in CD45-positive cells at the 0.1 mg/kg treatment group versus control (Figure 1A–C). This PCB dose-dependent increase was observed in both the epithelium and the stroma (Figure 1D,E). In addition, within the epithelium, there was an increase in CD45-positive cells at the 1 mg/kg treatment group compared to control (Figure 1D).

**Figure 2:**
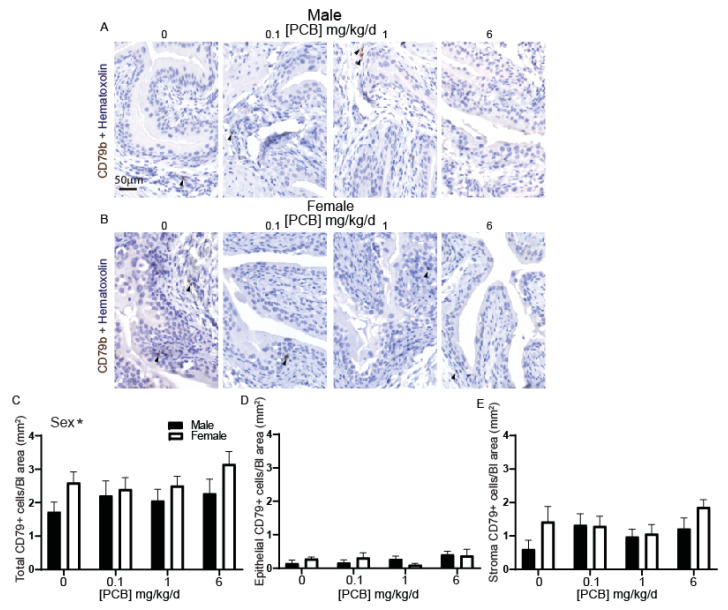
Sex influences CD79b-positive immune cells in bladders of developmentally exposed mice. Mice were exposed to PCBs via maternal diet through gestation and lactation. Bladders were collected from young male and female offspring at postnatal day (P) 28–31 for immunohistochemistry. Representative images of (**A**) male and (**B**) female mouse bladders at each PCB treatment group incubated with antibodies targeting CD79b (brown) to label immune cells and hematoxylin (purple) to label all nuclei. Quantification of (**C**) total cells CD79b positive within bladder, (**D**) epithelial cells CD79b positive within bladder, and (**E**) stromal cells CD79b positive within the bladder. Results are the mean ± SEM, *n* = 4–6 bladders per treatment group. * indicates significant differences at *p* < 0.05, as determined using two-way ANOVA.

**Figure 3:**
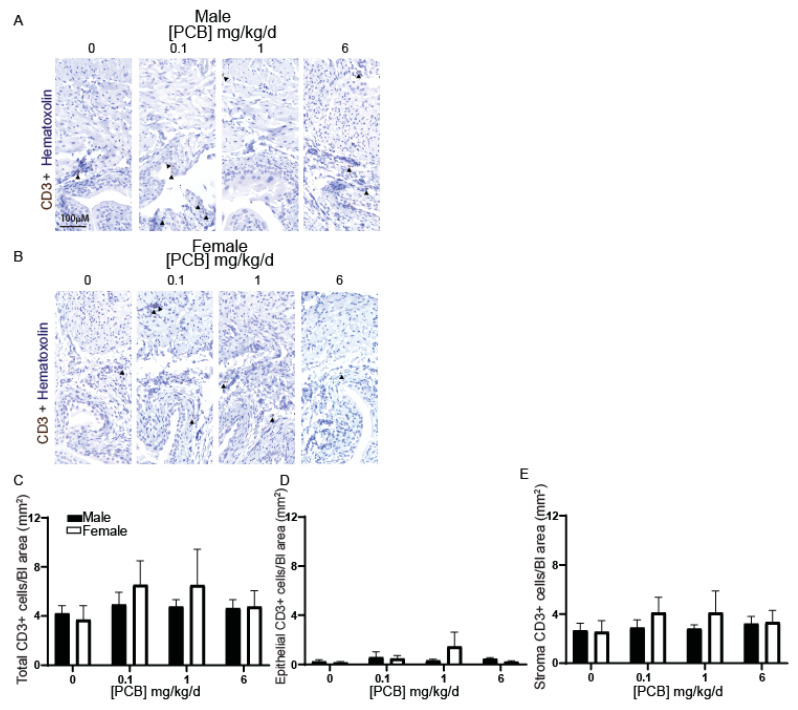
PCBs have no effect on CD3-positive immune cells in bladders of developmentally exposed mice. Mice were exposed to PCBs via maternal diet through gestation and lactation. Bladders were collected from young male and female offspring at postnatal day (P) 28–31 for immunohistochemistry. Representative images of (**A**) male and (**B**) female mouse bladders at each PCB treatment group incubated with antibodies targeting CD3 (brown) to label immune cells and hematoxylin (purple) to label all nuclei. Quantification of (**C**) the percent of total cells CD3 positive, (**D**) the percent of epithelial cells CD3 positive, and (**E**) the percent of stromal cells CD3 positive. Results are the mean ± SEM, *n* = 4–6 bladders per treatment group. No significant differences as determined by two-way ANOVA.

We next tested whether PCB exposure activates innate or adaptive immune responses within the bladder. We chose to focus on three immune cell populations which are dysregulated by PCBs in other tissues and are commonly dysregulated in patients with bladder dysfunction: B cells (adaptive), T cells (adaptive) [51,52] and macrophages (innate) [53,54]. B cells were immunolabeled with an antibody targeting CD79b (Figure 2A,B). PCB exposure did not significantly change the bladder abundance of CD79b-positive B cells; however, we identified a surprising overall main effect of sex with increases in total CD79b-positive cells in female compared to male bladder tissue (Figure 2C). PCB exposure did not change the number of CD79b-positive B cells within the epithelium or stromal cell types independently (Figure 2D,E). We visualized T cells using an antibody targeting CD3 (Figure 3A,B) and did not identify any significant sex- or dose-dependent differences within the bladder (Figure 3C–E). These results suggest that developmental PCB exposure does not activate the adaptive immune response within the bladder in juvenile mice.

**Figure 4:**
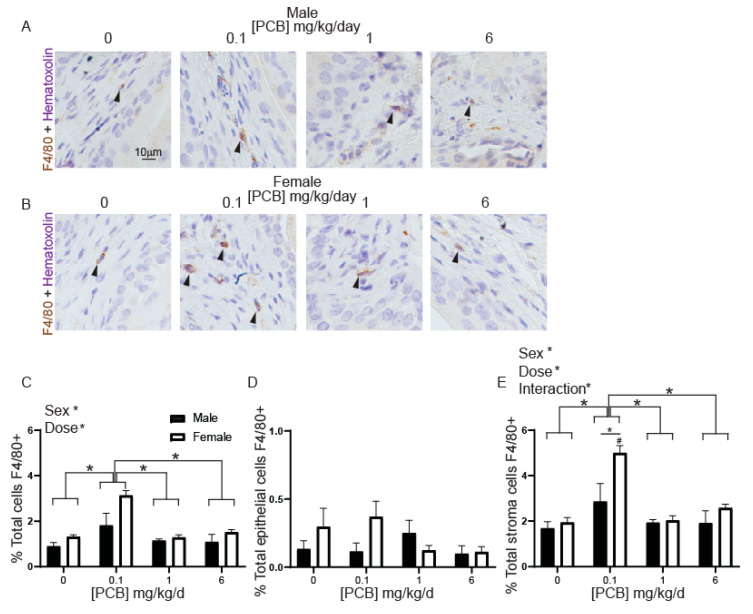
PCBs increase F4/80-positive cells in bladder of developmentally exposed female mice. Mice were exposed to PCBs via maternal diet through gestation and lactation and bladders collected from young male and female offspring at postnatal day (P) 28–31 for immunohistochemistry. Representative images of (**A**) male and (**B**) female mouse bladders at each PCB treatment group incubated with antibodies targeting F4/80 (brown) to label macrophages, nuclei were counterstained with hematoxylin (purple). Quantification of (**C**) percent total cells F4/80 positive, (**D**) percent total epithelial cells F4/80 positive, (**E**) percent total stromal cells F4/80 positive. Results are the mean ± SEM, *n* = 4–6 bladders per treatment group. Significant differences at *p* < 0.05 indicated by asterisk, asterisk and bars or by a # which indicates significant difference from all other same sex treatment groups as determined using two-way ANOVA followed by Tukey’s multiple comparisons tests.

Macrophages contribute to PCB-mediated inflammation in other tissues [53,55,56] and are mediators of the innate immune response. To test whether developmental PCBs altered this endpoint in our model, we examined F4/80-positive cells within the bladder as a marker of total macrophages (Figure 4A,B). There was a significant overall main effect of dose in the percentage of F4/80-positive macrophages, driven by a specific increase in the 0.1 mg/kg PCB treatment group compared to all other groups (Figure 4C). There was also an overall main effect of sex, with more macrophages in female than male bladders (Figure 4C). We did not observe a PCB-dependent change in the percentage of F4/80-positive cells within bladder epithelium alone (Figure 4D). However, in bladder stroma, there was a significant main effect of sex, dose and an interaction between sex and dose with F4/80-positive cells greater at the 0.1 mg/kg PCB treatment group compared to all other doses. This result was driven by a significant increase in F4/80-positive stromal cells in the female 0.1 mg/kg PCB treatment group compared to all other female treatment groups as well as between male and female at the 0.1 mg/kg PCB group (Figure 4E). These results indicate that the innate immune response can be activated by developmental PCB exposure which is especially evident at the lowest 0.1 mg/kg PCB group in female mice.

### 3.2. Developmental PCB Exposure Decreases Collagen Density within Female Bladder Stroma

**Figure 5:**
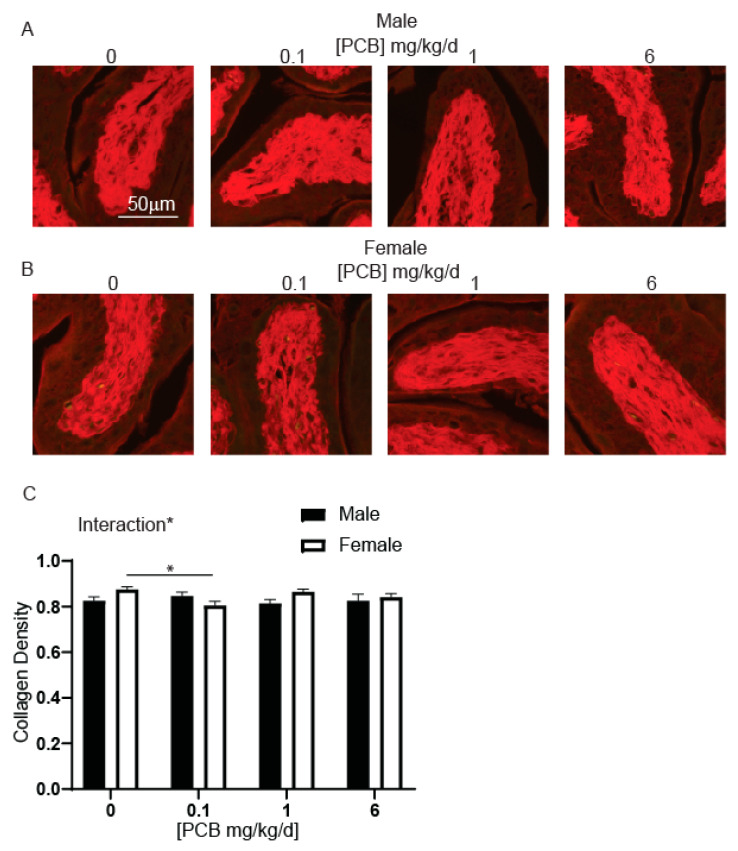
PCBs increase collagen density in bladder of developmentally exposed female mice. Mice were exposed to PCBs via maternal diet through gestation and lactation and bladders collected from young male and female offspring at postnatal day (P) 28–31. Slides were stained with picrosirius red to visualize collagen. Representative images of (**A**) male and (**B**) female mouse bladders at each PCB treatment group stained with picrosirius red to visualize collagen (red). Quantification of (**C**) ratio of collagen per unit area. Results are the mean ± SEM, *n* = 4–6 bladders per treatment group. Significant differences at *p* < 0.05 indicated by asterisk and asterisk and bar as determined using two-way ANOVA followed by Tukey’s multiple comparisons tests.

Chronic or acute inflammation can change collagen density [57] and PCBs have been linked to changes in collagen mRNA abundance and fiber density [58,59,60]. To test whether developmental PCB exposure changes collagen density in young mice, bladders were stained using picrosirius red (PSR) and stained fibers visualized under a Texas red filter (Figure 5A,B). There was a significant decrease in collagen density within bladder stroma in female mice in the 0.1 mg/kg/d PCB treatment group compared to control (Figure 5C). These results indicate that PCBs are capable of altering the extracellular matrix via changes in collagen density.

### 3.3. Developmental PCB Exposure Increases Proliferation in a Sex- and Dose-Specific Manner

**Figure 6:**
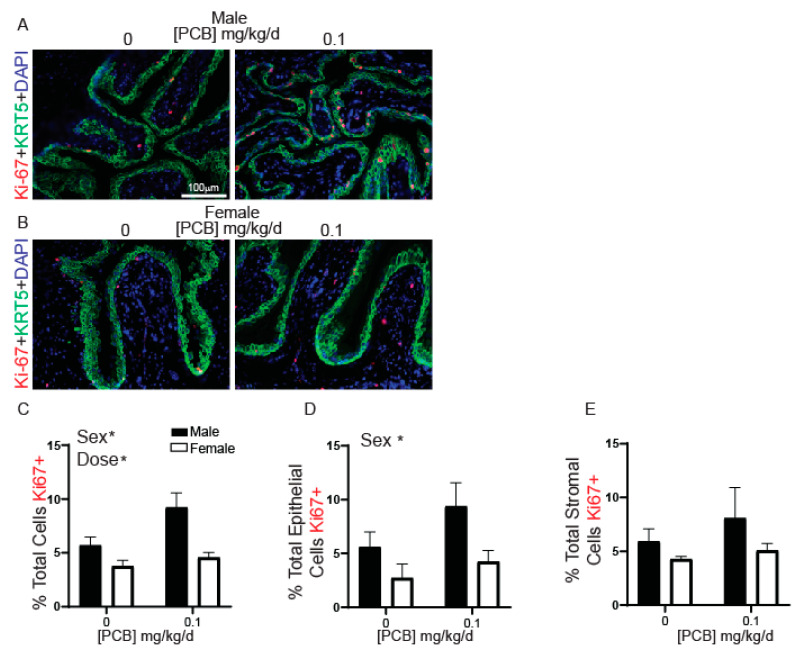
PCBs increase Ki-67-positive cells in male bladder. Mice were exposed to PCBs via maternal diet through gestation and lactation and bladders collected from young male and female offspring at postnatal day (P) 28–31 for immunohistochemistry. Representative images of (**A**) male and (**B**) female mouse bladders at each PCB treatment group incubated with antibodies targeting Ki-67 (red) to label proliferating cells, keratin 5 (KRT5, green) to label basal epithelium and DAPI (blue) to stain nuclei. Quantification of (**C**) the percent of total cells Ki-67 positive, (**D**) the percent of total epithelial cells Ki-67 positive and (**E**) the percent of total stromal cells Ki-67 positive. Results are the mean ± SEM, *n* = 4–6 bladders per treatment group, up to 3 images per bladder were averaged for final value. Significant differences at *p* < 0.05 indicated by asterisk as determined using two-way ANOVA followed by Tukey’s multiple comparisons tests.

The PCB-mediated (0.1 mg/kg) increase in the number of CD45+ hematolymphoid cells within bladder could indicate ongoing low-grade inflammation or a lingering response to a previous injury. Since proliferation is key to rapid recovery after injury to restore function [61,62], we used an antibody against Ki-67 to visualize cells in the active phase of the cell cycle to test whether PCB exposure at the 0.1 mg/kg dose increases bladder cell proliferation, which would be consistent with ongoing inflammation (Figure 6A,B). There was a significant overall main effect of dose and sex such that the percentage of Ki-67-positive bladder cells was greater in the 0.1 mg/kg PCB treatment group compared to control and was greater in male mice versus female mice (Figure 6C). When split into epithelial and stromal compartments significant effects were only observed in the epithelium (Figure 6D,E) where there was a significant main effect of sex with the percentage of Ki-67-positive cells greater in male versus female bladders (Figure 6D). Together, these results indicate that the 0.1 mg/kg PCB dose increases the number of cells in the active phase of the cell cycle most prominently in male mice.

## 4. Discussion

PCB exposure during in utero development and via lactation causes low-grade inflammation in the bladder and changes the cellular and molecular composition of the bladder in a dose- and sex-specific manner. The 0.1 mg/kg/d PCB dose increased the bladder concentration of CD45-positive hematolymphoid cells, macrophages and Ki67-positive cells and decreased collagen density in a sex-specific manner. These data suggest that innate immune responses can be evoked by low-dose PCB exposure during bladder development. We also found in female mice that PCBs increase the bladder’s density of macrophages, coincident with PCB-mediated changes to the bladder extracellular matrix. The impact of PCB-induced bladder inflammation on urinary function remains to be determined and is an area of future study. In addition, studies are needed to establish the timeline of inflammatory cell recruitment to the bladder of PCB exposed mice and the persistence of these cells into adulthood.

Increasing evidence suggests that exposure to environmental toxicants can contribute to lower urinary tract function in adulthood. Developmental exposure to environmental toxicant, dioxin (TCDD) via the dam, has been shown to decrease void intervals in adult male mouse offspring [63] and further exacerbates voiding dysfunction in susceptible mice [64,65]. There is also evidence that remodeling of collagen is one pathway by which environmental chemicals can impact the lower urinary tract. In rhesus monkeys, developmental TCDD exposure increases inflammatory cells and fibrosis in the prostate of offspring years after exposure [66]. In mice genetically susceptible to prostate neoplasia, developmental exposure to TCDD exacerbates hormone induced changes in collagen fiber size/distribution in the prostate and the bladder [65]. While these studies did not examine inflammation or presence of immune cells within the bladder, evidence that collagen fiber size and distribution were impacted by TCDD are in line with the results obtained here. We found a decrease in collagen density within female bladder of the 0.1 mg/kg/d PCB treatment group compared to controls, the same group which had an increase in F4/80-positive cells in the bladder. One possible explanation for this observation is that PCB exposure leads to increased degradation of the extracellular matrix and collagen, which is characteristic of some inflammatory disorders, such as asthma [57]. This could be due to the activity of macrophages, which produce matrix metalloproteinases (MMPs) that degrade extracellular proteins such as collagens [67]. Alternatively, PCBs may prevent the proper formation of collagen architecture within the bladder at this specific timepoint. This explanation is also supported by studies that found IL-1, an immune mediator released in response to stimuli such as allergens, suppresses the formation of collagen fibers [57], and that PCB contamination can lead to increased expression of mediators such as IL-1 [53]. Whether the observed decrease in collagen density is primarily due to collagen degradation or suppression of formation will require further research.

One factor which makes determining whether benign urologic diseases may have a developmental origin complex is the fact that dose-dependent effects are often seen with environmental chemical exposures. Non-monotonic PCB dose effects are commonly observed in the central nervous system [68,69]. Similarly, we saw increases in immune cells in the bladder in the lowest (0.1 mg/kg) dose group but not in higher dose groups. It can be difficult to compare dose effects across studies when different ages and dosing paradigms are used, nonetheless our findings are consistent with other PCB immune studies. For example, only a low single dose (20 mg/kg) exposure to Aroclor 1260 in adult male mice elevated serum IL-6 in response to a high-fat diet weeks later; higher concentrations of Aroclor 1260 (200 mg/kg) did not [70]. This suggests a dose-specific effect of PCBs on inflammatory markers, as well as the ability of PCBs to interact with other factors such as diet [70]. One explanation for PCB dose effects could be that higher doses of PCBs lead to toxicity and cell death. However, apoptosis is not a likely factor in the lack of response observed in the higher PCB dose groups here. Our previous study in mice dosed in the same manner and of the same age, had detectable levels of PCBs within bladder tissue, but did not display any changes in cleaved caspase 3 apoptotic cells in the bladder, changes in epithelial composition or thickness, or any changes in mouse body mass or urine creatinine concentration [32]. A second explanation for dose effects observed is that low versus high doses of PCBs could produce a different trajectory of changes within the bladder. It is possible that the higher PCB doses elicited an inflammatory response at an earlier timepoint than was examined. On the other hand, it is also possible that each dose triggers a different set of receptors/pathways which contribute to bladder phenotypes. We have previously observed PCB induced increases in nerve fiber density within the male bladder only in the higher 6 mg/kg PCB group [32]. Whether this increase in nerve density was preceded by inflammation or arose independently of an inflammatory event remains to be determined. Dose effects could also be due to differences in metabolizing enzymes present, the effects of the metabolites themselves on the tissue, or differences in receptor targets.

We identified an increase in macrophages within bladders of mice exposed to the lowest PCB dose which was prevalent in female offspring. This finding is consistent with previous studies which have examined effects of PCBs on serum cytokine and chemokine expression [45]. Macrophages are capable of secreting various cytokines including tumor necrosis factor (TNF), interleukin (IL)-1, IL-6, IL-8, IL-12, IL-23 as well as chemokines such as CXCL1 and 2, CCL5, CXCL8-11 [71]. In a study using the same dosing paradigm used here, PCBs elicited dose-dependent increases in several serum cytokines and chemokines including TNFa, IL-1a, IL-1b, IL-10, GM-CSF, IL-17, IL-12, IFN-g, IL-4, IL-9, IL-13, CCL3, and CXCL1 [45]. While not all chemokines were examined, the PCB effect on increasing serum TNFa, IL-1, IL-12 and CXCL1 are consistent with the hypothesis that macrophages may be involved in PCB induced inflammatory responses in the bladder and perhaps other tissues. In addition to serum cytokines, another study using the same dosing paradigm used here reported increases in IL-6 and IL-1b in intestine of developmentally exposed mice [44]. Exposure to PCB 126 (50–500 nM) in macrophage cell lines induced expression of proinflammatory cytokines such as monocyte chemoattractant protein-1 (MCP-1) which is involved in macrophage recruitment [53]. Epidemiological data from Inuits with high PCB consumption report a significant increase in serum levels of inflammatory markers, YLK-40 and hsCRP [17]. Elevation of YKL-40 is of particular interest as it can be secreted by macrophages [72]. Together, these results are in line with increased macrophages in PCB exposed bladder observed here and suggest that macrophages may play an important role in PCB-induced changes in inflammation, especially in female bladder.

Macrophage polarization can lead to differences in response to stimuli and can contribute to sex differences in inflammatory responses. For example, in a myocarditis rat model, males tend to exhibit a M1 driven pro-inflammatory response while females predominantly exhibit an M2 anti-inflammatory response [73]. These states of macrophage polarization also correspond to severity of fibrosis. Males had increased expression of collagen gene transcripts and fibrosis while females had upregulated expression of anti-fibrotic genes and downregulation of pro-fibrotic genes [73]. It is possible that the observation here of increased macrophages in female bladder in the 0.1 mg/kg PCB treatment group that also had decreased collagen density, is due to a mechanism related to macrophage polarization. Whether PCBs alter the polarization of macrophages, and whether the macrophages present in the female bladder were M1 or M2 and linked to changes in expression of genes which are pro- or anti-fibrotic in nature, remains an area of future study.

The presence of increased CD45-positive cells in the bladder suggests that one possible mechanism of action for developmental PCB exposure on the bladder is setting up a state of low-grade inflammation. This may persist or be cleared, but it raises the possibility that PCBs could act as a stressor causing a low-grade inflammatory reaction ongoing in the bladder. This could lead to increased sensitivity to other stressors throughout life especially if those stressors also trigger inflammation. The observed increase in macrophages in female bladder is also intriguing. In humans, macrophages are known to play a role in response to urinary tract infection which is more prevalent in women compared to men [74,75,76]. Women are also more commonly affected by interstitial cystitis/bladder pain syndrome than men, and it is thought that a component of this diagnosis is underlying bladder inflammation [3,77]. Whether environmental exposures to PCBs can contribute to the etiology or sex biases of these disorders is unknown, but of potential clinical interest as steps to reduce exposure, especially during development, could help reduce risk or severity of these lower urinary tract symptoms later in life.

## Figures and Tables

**Table 1:** List of Antibodies.

Primary Antibodies	Catalog #	Company	Source	Dilution	Pairing
CD79b	ab134147	Abcam	Rabbit	1:250	
CD3	ab11089	Abcam	Rat	1:250	
CD45 (PTPRC)	ab10558	Abcam	Rabbit	1:750	
E-Cadherin (CDH1)	610181	BD Transduction Labs (via Fisher)	Mouse	1:250	
F4/80 (EMR1; Adgre1)	123102	Biolegend	Rat	1:50	
Ki67	ab15580	Abcam	Rabbit	1:200	
Keratin 5	905901	Biologend	Chicken	1:500	
Secondary Antibodies/Detection Kits					
Anti-Mouse Alexa Fluor 594	715-545-150	Jackson ImmunoResearch	Donkey	1:250	Cdh1
Anti-Rabbit Alexa Fluor 488	711-545-152	Jackson ImmunoResearch	Donkey	1:250	CD45
Anti-Rabbit Alexa Fluor 594	111-585-144	Jackson ImmunoResearch	Goat	1:250	Ki67
Anti-Rat Biotinylated	112-066-003	Jackson ImmunoResearch	Goat	1:250	CD3, F4/80
Anti-Chicken Alexa 488	703-546-155	Jackson ImmunoResearch	Donkey	1:250	Krt5
ImmPRESS^®^ HRO Horse Anti-Rabbit IgG Polymer Detection Kit, Peroxidase	MP-7401	Vector Laboratories	Horse	Per manufacturer’s instructions	CD79b
VECTASTAIN ELITE HRP ABC KIT paired with DAB substrate Kit, Peroxidase	PK-6100, SK-4100	Vector Laboratories		Per manufacturer’s instructions	CD3, F4/80

## Data Availability

Available upon request to corresponding author.
